# Response to High‐Dose Topical Capsaicin in Neuropathic Pain: Absence of Deep Pain as a Predictor of Analgesic Effect

**DOI:** 10.1002/ejp.70200

**Published:** 2026-01-09

**Authors:** Manon Sendel, Lena Ehmke, Andreas Dunst, Jan Vollert, Henrike Bruckmüller, Sandra Brügge, Stefanie Rehm, Janne Gierthmühlen, Dilara Kersebaum, Ingolf Cascorbi, Ralf Baron, Julia Forstenpointner

**Affiliations:** ^1^ Division of Neurological Pain Research and Therapy, Department of Neurology University Hospital Schleswig‐Holstein Kiel Germany; ^2^ Department of Clinical and Biomedical Sciences, Faculty of Health and Life Sciences University of Exeter Exeter UK; ^3^ Institute for Experimental and Clinical Pharmacology University Hospital Schleswig‐Holstein Kiel Germany; ^4^ Department of Pharmacy UiT The Arctic University of Norway Tromsø Norway; ^5^ Department of Gynecology and Obstetrics University Hospital Schleswig‐Holstein Kiel Germany

## Abstract

**Background:**

Topical high‐dose capsaicin treatment is a recommended therapy for localised neuropathic pain with good tolerability and no identified drug interactions. However, it is not effective for every patient. So far, few parameters in patient history have been identified as predictors for response to capsaicin treatment. The aim of this prospective non‐interventional exploratory study was to improve prediction of treatment response to high‐dose capsaicin by including quantifiable parameters and standardised questionnaires.

**Methods:**

Forty‐eight patients with peripheral neuropathic pain were included in the study. In addition to questionnaires assessing pain and patient‐reported outcome measures, the function of transient receptor potential vanilloid 1 (TRPV1) receptors in the affected skin was assessed via functional laser‐speckle‐contrast‐analysis (fLASCA) and sensory testing was performed. Furthermore, the effects of genetic variants in TRPV1 and endothelial NO‐synthase (eNOS) were tested by genotyping for TRPV1 rs8065080 (c.1911A>G, p.I585V), TRPV1 rs222747 (c.1103C>G, p.M315I) and eNOS rs1799983 (c.894 T>G, p.D298E).

**Results:**

Patient‐reported catastrophizing was identified as the most important response predictor. Higher vibration detection threshold and higher pressure pain threshold showed the highest prediction values of sensory parameters. Combined, these three parameters predicted over a quarter of the level of pain relief. The genetic variant for TRPV1 rs222747 showed a significant impact on pain relief with a pain relief prediction of 13% or more.

**Conclusion:**

A higher pressure pain threshold, a higher vibration detection threshold, higher pain catastrophizing and the presence of the TRPV1 variant rs222747 are associated with more pain relief from high‐dose capsaicin treatment and provide promising targets for future investigation.

**Significance Statement:**

This exploratory study identifies promising predictors for analgesic response to high‐concentration capsaicin. The models generated in this study include a spectrum of different variables considering psychological factors as well as functional nerve‐fibre assessments and genetic polymorphisms.

## 1 Introduction

Capsaicin acts as an agonist at the TRPV1‐receptor located on primary afferent nociceptive neurons. While low‐dose capsaicin induces a sensitization to further capsaicin stimuli within hours, high‐dose capsaicin application can lead to degeneration of nociceptive fibres and analgesia lasting for several weeks (Arora et al. 2021). In localised peripheral neuropathic pain, a single 30–60‐min application of high‐concentration capsaicin can lead to analgesia for up to three months. Treatment is generally well tolerated (Gálvez et al. 2017).

However, not every patient benefits from the treatment. In some cases, several applications are needed to reach an analgesic effect, while some do not benefit at all (Freynhagen et al. 2021; Soliman et al. 2025).

Prediction of response helps the proper allocation of resources and to minimise burden on patients.

Martini et al. (2012) identified different response groups in a cohort of 91 patients with diabetic neuropathy, who received a single treatment with high‐concentration capsaicin and linked those to different pathomechanisms. Demographic parameters such as sex, age or pain duration have been described as predictors for response to capsaicin treatment (Arora et al. 2021; Martini et al. 2012). However, further improvement of response prediction models might lead to streamlining of therapy options (Baron et al. 2023).

Recently, high‐concentration capsaicin treatment has been linked to nerve fibre regeneration (Anand et al. 2019, 2021, 2022; Sendel et al. 2023). One study showed that patients exhibiting functional recovery in terms of heat‐evoked neurogenic vasodilatation experienced a greater reduction in pain from treatment than those who did not (Sendel et al. 2023). This raises the question of whether the function of peptidergic C‐fibres before treatment may have an influence on treatment response.

In healthy subjects, capsaicin‐induced sensory changes vary depending on genetic variants in the TRPV1‐receptor (Forstenpointner et al. 2017; Kringel et al. 2018), thereby suggesting a possible impact of genetic variants on treatment efficacy.

Additionally, TRPV1 is closely linked with the enzyme catalysing production of nitric oxide (endothelial NO‐synthase, eNOS) to induce vasodilatation. eNOS is regarded as a driver of nerve revascularization (Keilhoff et al. 2003) as it is found to be overexpressed in vasa nervorum soon after experimental nerve injury (González‐Hernández and Rustioni 1999). However, too high NO concentrations seem to hinder axonal regrowth (Henrich et al. 2004; Hess et al. 1993; Moreno‐López 2010).

The eNOS missense variant rs1799983 (c.894 T>G, p.D298E) is not only associated with cardiovascular disorders but also with an increased risk of neuropathy in patients with diabetes mellitus (Costacou et al. 2006).

Regardless, the impact of genetic variants on response to treatment with capsaicin has not been thoroughly investigated so far.

In this study, patients with peripheral neuropathic pain were analysed aiming to further improve the precision of predicting the response to high‐concentration capsaicin by including quantifiable parameters and using standardised questionnaires.

The study aimed to examine whether the response to treatment (pain relief and/or improvement in quality of life; QoL) was associated with the following parameters assessed prior to treatment initiation: (1) afferent fibre function and TRPV1 receptor activity, (2) common genetic variants in the TRPV1 and eNOS genes and (3) results from validated questionnaires.

## 2 Methods

### 2.1 Study Design

In this non‐interventional exploratory trial, 48 patients (22 males/26 females) with peripheral neuropathic pain were included who were scheduled to be treated with topical high‐concentration capsaicin (8%) as part of their treatment regimen.

The inclusion criteria were men or women with peripheral neuropathic pain aged ≥ 18 years. The exclusion criteria were severe depression, chronic alcoholism, substance abuse or any reason preventing an accurate understanding of the tests. Prior use of systemic analgesic medication could be continued during the study.

Patients were recruited between 2018 and 2021. All patients were capsaicin‐naïve before treatment initiation. The patients suffered from peripheral neuropathic pain of different etiologies (e.g., painful polyneuropathy, peripheral nerve injury, or postherpetic neuralgia) and were recruited from the pain therapy facilities (Departments of Anesthesiology and of Neurology and the Gynaecological Cancer Center of the University Hospital Schleswig‐Holstein, Campus Kiel, Germany) as well as from associated outpatient centers.

One patient was excluded because of incomplete baseline data.

At baseline (before the planned treatment with topical high‐concentration capsaicin) the functional integrity of the TRPV1 receptors in the affected skin was assessed via functional laser‐speckle‐contrast‐analysis (fLASCA) and quantitative sensory testing (QST) was performed. Capsaicin was applied regardless of fLASCA and QST results.

All tests were performed directly before the first capsaicin application.

A set of questionnaires was completed by patients at baseline, including (i) the mean, maximum and minimum intensity of their pain during the last 24 h on an 101‐point (0–100) numerical scale (NRS), (ii) the painDETECT questionnaire including a drawing of painful areas, (iii) the Neuropathic Pain Symptom Inventory (NPSI), (iv) the Pain Catastrophizing Scale (PCS), (v) the Pain Sensitivity Questionnaire (PSQ), (vi) the Mainz Pain Staging System (MPSS), (vii) the Hospital Anxiety and Depression Scale (HADS), (viii) and a questionnaire to capture quality of life and functional disability (SF‐12). Furthermore, the duration of the disease and the duration of the pain syndrome were assessed.

Additionally, blood samples were drawn for genetic analyses of single nucleotide variants (SNV) within the TRPV1 and eNOS coding genes.

Treatment was performed using 8%‐capsaicin patches (Qutenza 179 mg, Grünenthal GmbH, Germany) as recommended by the manufacturer.

Thirty days after topical high‐concentration capsaicin application, a set of questionnaires, including NRS ([0–100], maximum/minimum/average pain), SF‐12 and PGIC (Patient Global Impression of Change), was completed.

NRS and PGIC were also assessed after 2, 10 and 12 weeks via telephone interview.

The study was approved by the local ethics committees. Patients received information material on all study procedures including information on genetic testing and the handling of the acquired data. Informed written consent was obtained from all participants according to the Declaration of Helsinki. The study was registered at DRKS (Deutsches Register Klinischer Studien; DRKS00016611).

### 2.2 Functionality of Peptidergic Nociceptors

To determine the peripheral function of peptidergic nociceptors, fLASCA was used to assess changes in the microcirculation via the PeriScan PIM 3 system (laser‐Doppler perfusion imager; Perimed, Stockholm, Sweden). The technical set up was identical to a previously conducted study (Forstenpointner et al. 2020). Peripheral TRPV1‐positive peptidergic C‐fibres were activated by continuous heating (42°C) of a circular thermoprobe (probe 415‐339, Perimed, Stockholm, Sweden), which was attached to the affected skin areas. The measurements were conducted within a timeframe of 40 min, consisting of a 5‐min adaptation phase without any intervention, a 10‐min baseline phase in which the skin was heated to 32°C, followed by a 25‐min period of heating the skin up to 42°C. To reduce biological variations in skin perfusion, all measurements were performed in a climatized quiet room in supine position. The patients were asked to refrain from smoking and caffeine intake at least 4 h before the experiments. To reduce individual heterogeneity in baseline fLASCA measurements, the statistical analysis compares differences of relative changes in blood perfusion.

### 2.3 Quantitative Sensory Testing

The nerve fibre function of different groups of peripheral and central afferent pathways was measured psychophysically. QST was conducted according to the protocol of the German Research Network on Neuropathic Pain (Rolke et al. 2006). This protocol assessed the function of small and large fibres and their central pathways with particular interest towards the function of small fibres, that is, thermal and pain perception thresholds. For evaluation of individual QST measurements, data were compared to a reference database of healthy controls and *z* scores were calculated. The calculation of *z* values indicated hypo‐ or hypersensitivity of each sensory parameter and allowed correction for age‐, gender‐ and localization‐dependent alterations of sensory function. The 95% confidence interval of controls is between −1.96 and +1.96. Positive *z* values indicate hyperfunction, that is, patients are more sensitive to the tested parameter compared to controls (lower thresholds), whereas negative *z* values indicate hypofunction, that is, a loss or lower sensitivity towards a stimulus as compared to controls (higher thresholds).

Testing sites have been specified in Table S2.

### 2.4 Genotype Analysis

Genomic DNA of all patients was extracted from venous blood samples using the Gentra Puregene Blood Kit (Qiagen, Hilden, Germany) according to the manufacturer's instructions. The frequent TRPV1 gene variants rs8065080 (c.1911A>G) and rs222747 (c.1103C>G) were genotyped by pyrosequencing using a PyroMark Q48 (Qiagen, Hilden, Germany). The PCR was performed according to the protocol of Meridian Biosciences (Memphis, USA) for MyTaq Mix with 1 μL gDNA, 10 μmol of respective forward and reverse primers and MyTaq Mix 2× (Table S1). PCR reactions were executed at 95°C for 1 min, 35 cycles containing 15 s at 95°C, 15 s annealing at 63°C and 10 s elongation at 72°C. Final elongation was executed for 4 min at 72°C. Pyrosequencing with PCR product was conducted according to the manufacturer's recommendations. eNOS rs1799983 (c. 894 T>G) was analysed with TaqMan allelic discrimination assay C_3219460_20 (ThermoFisher Scientific, Darmstadt, Germany) on an ABI Prism 7900 HT device according to the manufacturer's protocol.

### 2.5 Statistical Analysis

If not stated otherwise, the parameters are displayed as mean ± standard deviation.

Due to non‐normal distribution according to the Shapiro–Wilk test and relatively small sample size, non‐parametric tests were used. To test differences between groups, the Mann–Whitney *U*‐test was applied. Comparisons in between timepoints were performed via the Wilcoxon signed rank test. Spearman's rank correlation was used for correlation analysis. *χ* ^2^ test was used for calculation of differences in categorical data. All calculations were performed with SPSS Statistics 27.0 (IBM, Chicago, IL, USA). *p* values < 0.05 were considered statistically significant.

#### 2.5.1 Prediction Models

To identify the influence of baseline parameters on treatment response (defined as reduction in mean pain in the last 24 h or increase of quality of life as assessed by SF‐12), appropriate variables were chosen via forward stepwise regression. All available baseline characteristics are listed in Table 1.

Three regression model analyses were performed to investigate the potential to predict individual patient level response to high‐concentration capsaicin, with pain improvement from baseline to week four and improvement of quality‐of‐life (SF‐L12) as outcome measures. The first of these regression analyses applied a data‐driven approach; the other two were hypothesis‐driven: (1) All available baseline parameters were included (2) The two genetic variants of the TRPV1 receptor and the variant in eNOS were set to be included (3) The functional integrity of nociceptors as analysed with the fLASCA was included into the model as a baseline parameter. Patients were only included in the models when all required data was available. In case of a significant model, we constructed three similar models with alternative outcomes as sensitivity analyses: mean pain reduction at week 12, maximum pain reduction at week 4 and maximum pain reduction at week 12 and provide explained variance *r* ^2^ for these along with the main model.

#### 2.5.2 Model Selecting From All Baseline Parameters

For this model, we applied a data‐driven, forwards‐stepwise approach to develop a prediction model for the endpoints pain intensity and change in quality of life after 4 weeks with all baseline variables available. The most suitable variable for prediction was added until the model could not be improved further by adding additional variables.

These included demographic variables (age, sex, diagnosis), baseline pain intensity, painDETECT score, pain during capsaicin application, pain catastrophizing upon pain catastrophizing scale, QST parameters (vibration detection threshold, pressure pain threshold, mechanical pain threshold, warmth detection threshold, heat pain threshold, wind‐up ratio and dynamic mechanical allodynia), genetic variants (TRPV1 rs222747 and rs8065080, eNOS rs1799983 assuming an additive genetic model), as well as NO‐ and CGRP‐peaks measured via fLASCA in affected and control skin area.

Thirty‐nine patients could be included. Baseline parameters are listed in Table 1. There is no significant difference to the overall group.

#### 2.5.3 Model Based on Genetic Polymorphisms

In this approach the two candidate TRPV1 polymorphisms as well as the eNOS polymorphism were included and set to stay in the model assuming an additive genetic model was tested. Datasets from all 48 patients were available for analysis. Allele frequencies as well as Hardy–Weinberg equilibrium are depicted in Table 2.

**TABLE 1:** Patient characteristics of all 48 assessed patients as well as of the 39 patients with available fLASCA data.

	All 48 assessed patients	39 patients with available fLASCA data
Age (years)	61.7 ± 16.0	64.0 ± 15.2
Sex (male/female) (*n*)	22/26	18/11
Diagnosis (*n*)		
Polyneuropathy	23	19
Peripheral nerve injury	15	10
Postherpetic neuralgia	9	9
Notalgia paraesthetica	1	1
30‐min application (feet only) (*n*)	16	13
Duration of pain (months)	35.4 ± 33.6	36.0 ± 34.1
Mainz pain staging system score	15.6 ± 3.3	15.6 ± 3.2
Pain sensitivity questionnaire	62.2 ± 21.1	61.6 ± 22.2
Pain catastrophizing Scale (total)	21.2 ± 10.4	21.2 ± 10.6
Neuropathic pain symptom inventory (total)	17.2 ± 9.9	16.7 ± 9.5
HADS anxiety	5.9 ± 4.1	6.1 ± 4.1
HADS depression	6.6 ± 4.4	6.8 ± 4.1

*Note:* Parameters displayed as mean ± standard deviation, if not stated otherwise.

Abbreviations: fLASCA, functional Laser Speckle Contrast Analysis; HADS, Hospital Anxiety and Depression Scale.

#### 2.5.4 Baseline Parameter: Functional Integrity of the Peripheral TRPV1 Receptor in the Painful Skin

For model 3, the function of vasoactive C‐fibres (baseline measurement of fLASCA/CGRP‐peak as well as NO‐peak) was included in the model (hypothesis I). We tested the influence of this baseline parameter on pain relief at week four. As in the model with all included parameters, 39 patients could be included. For the remaining nine patients, perfusion data was not available due to insufficient quality of raw data or due to problems scheduling in‐person appointments due to the Covid‐19 pandemic.

Various models using multiple estimator methods with and without interaction term were attempted.

## 3 Results

### 3.1 Patient Characteristics

Patient characteristics at baseline are displayed in Table 1.

**TABLE 2:** Investigated SNVs and allele frequencies.

rsID	Gene	Common variant name	Function	Maj>Min	MAF	Hardy–Weinberg equilibrium	
						Heterozygous	Homozygous
rs8065080	TRPV1	c.1911 T>C, I585V	Missense	A>G^a^	0.386	0.470/0.458 *p* = ns	0.151/0.188 *p* = ns
rs222747	TRPV1	c.1103C>G, M315I	Missense	C>G^a^	0.250	0.372/0.396 *p* = ns	0.064/0.040 *p* = ns
rs1799983	eNOS	c.894 T>G, D298E	Missense	T>G	0.681	0.416/0.312 *p* = ns	0.472/0.458 *p* = ns

Abbreviations: MAF, minor allele frequency; Maj, major allele; Min, minor allele.

^a^ Strand flip compared to common variant name.

### 3.2 Efficacy of High‐Concentration Capsaicin Treatment

One‐time topical treatment with high‐concentration capsaicin in the entire cohort (*n* = 48) led to a significant reduction of the maximum pain intensity in comparison to baseline during the last 24 h at all time points (2, 4, 10, 12 weeks, *p* ≤ 0.01) and improved the quality of life (SF 12, assessed at baseline and week four, *p* = 0.043) of the patients significantly. Mean change in mean and minimum pain intensity during the last 24 h did not reach statistical significance (Table 3).

**TABLE 3:** Efficacy of capsaicin treatment.

	Baseline (*n* = 47)	2 weeks (*n* = 47)	*p*	4 weeks (*n* = 45)	*p*	10 weeks (*n* = 45)	*p*	12 weeks (*n* = 43)	*p*
Mean pain (24 h)	40.1 ± 18.8	36.7 ± 25.9	ns	36.7 ± 22.3	ns	37.0 ± 25.4	ns	37.6 ± 23.4	ns
Least pain (24 h)	18.0 ± 18.4	16.7 ± 20.9	ns	21.0 ± 23.0	ns	19.4 ± 22.5	ns	21.4 ± 20.7	ns
Maximum pain (24 h)	69.5 ± 22.2	59.0 ± 24.8	0.002	56.1 ± 26.8	< 0.001	55.0 ± 28.8	< 0.001	58.1 ± 26.6	0.001
SF 12 (overall)	40.1 ± 18.8	n/a	n/a	41.8 ± 8.4	0.043	n/a	n/a	n/a	n/a

Abbreviations: n/a, not assessed; SF 12, short‐form symptom inventory 12 questions.

### 3.3 Influence of Polymorphisms on Perfusion Measurements

No significant difference between carriers and non‐carriers of the investigated polymorphisms was observed in mean change in CGRP‐ and NO‐induced perfusion between baseline and Week 4.

### 3.4 Influence of Genetic Variants on Discomfort During Application

No significant difference between carriers and non‐carriers of the investigated polymorphisms was observed in pain or heat sensation during capsaicin application.

### 3.5 Prediction of Response to Topical High‐Concentration Capsaicin Treatment

We constructed three different prediction models for response to capsaicin treatment. While we chose a stepwise approach in model one, the other two models were compiled using a hypothesis‐driven approach. None of the models were able to identify predictors for quality of life as an outcome.

#### 3.5.1 Model Selecting From All Baseline Parameters

The resulting model for pain reduction as an outcome included three predictors (in decreasing order of importance): pain catastrophizing (*β* = 0.503, *p* < 0.001), vibration detection threshold (β = −0.302, *p* = 0.026), pressure pain threshold (β = −0.214, *p* = 0.109).

The full model function is given as follows:

$$\text{Pain change}=-11.410+0.952\;\left(\mathrm{PCS}\right)-4.298\;\left(\mathrm{VDT}\right)-1.605\;\left(\mathrm{PPT}\right).$$

**FIGURE 1:**
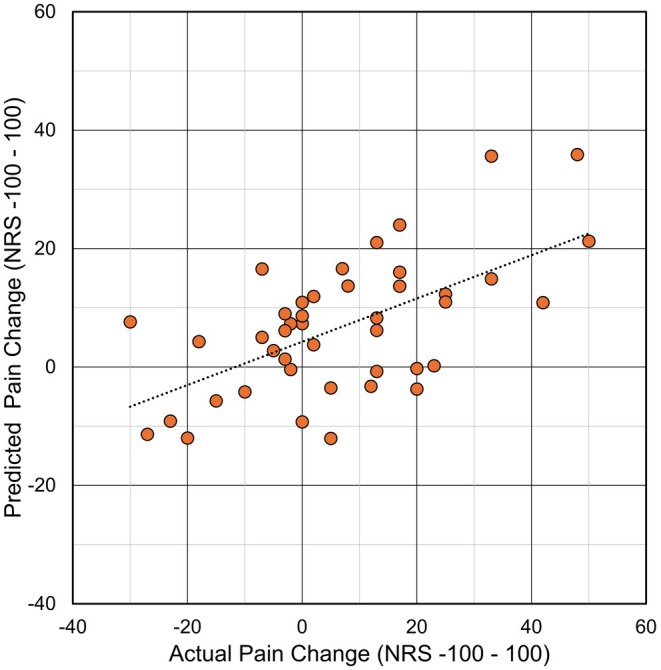
Comparison of actual and predicted change in pain for the model including all baseline parameters.

The model reached an accuracy in prediction (explained variance *r* ^2^ between observed and predicted change from baseline) of 26.6% (equivalent to a correlation coefficient *r* = 0.51; *F* = 7.465; df_regression_ = 3; df_residual_ = 39, *p* < 0.001; Figure 1). High pain catastrophizing as well as low perception of vibration and pressure pain were associated with a high response. For the three sensitivity analyses, this model provided the following explained variance: mean pain reduction at week 12, *r* ^2^ = 0.13; maximum pain reduction at week 4, *r* ^2^ = 0.31; maximum pain reduction at week 12, *r* ^2^ = 0.22.

#### 3.5.2 Model Based on Genetic Variants

While the overall model did not reach statistical significance with a *p* value = 0.073, TRPV1 rs222747 showed a significant impact (*p* = 0.028) on pain relief (for details see Table 4).

**TABLE 4:** Statistics of prediction model including genetic variants.

	Type III sum of squares	df	Mean square	*F*	*p* value
Corrected model	4034.917	7	576.4	2.0	0.073
Intercept	3488.856	1	3488.9	12.4	0.001
TRPV1 rs222747	2203.843	2	1101.9	3.9	0.0
TRPV1 rs8065080	918.604	2	459.3	1.6	0.209
TRPV1 rs222747 × TRPV1rs8065080	1653.640	3	551.2	2.0	0.137
Error	11,291.414	40	282.3		
Total	16,863.889	48			
Corrected total	15,326.331	47			

Abbreviation: df, degrees of freedom.

The overall variance explained by the model totals up to an estimated 26% (*r* ^2^ = 0.263; *F* = 2.042; df_regression_ = 7; df_residual_ = 40, *p* < 0.001). As expected in the additive model, homozygous minor allele was associated with the highest capsaicin response, compared to heterozygous and homozygous major allele.

No model could be improved by adding the eNOS polymorphism as a variable.

#### 3.5.3 Baseline Parameter: Functional Integrity of the Peripheral TRPV1 Receptor in the Painful Skin

Using this model, no significant predictors could be identified neither for pain intensity nor for quality of life as an outcome measure.

## 4 Discussion

In this study, prediction models using baseline parameters as well as predefined genetic variants in the TRPV1 gene were generated using data of a cohort of patients with neuropathic pain of different etiologies. Reduction in mean pain in the overall group was not significant and the effect on maximum pain was moderate, which is in line with high reported numbers needed to treat (Soliman et al. 2025).

The models revealed four significant predictors for treatment response regarding pain reduction: vibration detection threshold and pressure pain threshold in the affected area assessed with QST, pain catastrophizing and the TRPV1 variant rs222747 (c.1103C>G, p.M315I).

### 4.1 Deep Somatic Input

Different QST parameters have been identified as possible indicators of response to capsaicin treatment in previous trials. Among them are absence of allodynia or presence of cold and pinprick hyperalgesia (Arora et al. 2021; Mainka et al. 2016) which could not be identified as predictors of response to capsaicin in our study. A possible explanation for the different results could be the heterogeneity of the study cohorts.

In line with our results, the study by Gustorff et al. (2013) already showed that responders to capsaicin have a significantly lower pressure pain threshold in an area affected by neuropathic pain compared to a control area before therapy.

The pressure pain threshold is mainly a marker of nociceptive innervation of deep somatic tissues. We found that deep somatic hyperalgesia is associated with a low response to capsaicin treatment. This very likely is a generalised predictive effect which is applicable to all topical medications. If an important nociceptive input from deep tissues is present as indicated by deep hyperalgesia, a topical medication is less likely to work.

Several lines of reasoning indicate that deep somatic nociceptive input to the spinal cord is even more important to induce central sensitization and chronicity than cutaneous input (Wall and Woolf 1984). In polyneuropathies, postherpetic neuralgia and complex regional pain syndromes, 5%, 39% and 66% of patients have abnormally low‐pressure pain thresholds, respectively (Maier et al. 2010). Thus, patients with polyneuropathies should theoretically benefit most from topical medications. However, in an open label study, the effect was comparable between different peripheral neuropathic pain syndromes (Maihofner and Heskamp 2013).

Furthermore, a reduced sensation of vibration was found to predict a good response. Like pressure pain, the sensation of vibration is mainly conveyed by subcutaneous/deep somatic afferents and only to a minority by cutaneous nerves. It seems plausible that a topical therapeutic approach only targeting cutaneous nerves is more effective if there is less deep somatic sensory input.

### 4.2 Pain Catastrophizing

Patient‐reported catastrophizing was found to be the most important predictor. Even in a singular simple linear regression model only including pain catastrophizing, it accounted for 17% explained variance (*p* = 0.004). Interestingly, a higher score upon the pain catastrophizing scale predicts a high response to high‐concentration capsaicin.

Across different interventions, pain catastrophizing emerges as a strong predictor of pain treatment outcomes, in general being associated with negative phenomena. In general, pain catastrophizing is associated with worse pain outcomes regardless of the intervention applied (Baxter et al. 2022; Racine et al. 2016; Wertli et al. 2014). One explanation why our study shows different results may be the lack of a placebo control, as some studies show patients exhibiting higher pain catastrophizing to experience greater pain relief through placebo than those with lower catastrophizing (Sullivan et al. 2008; Weng et al. 2022).

This could also be expression of a generalised predictive effect indicating that patients with high catastrophizing scores tend to prefer and therefore respond better to topical medications because of their fear of systemic side effects (Schubert et al. 2021). It has been shown that local therapy with capsaicin 8% patch is non‐inferior to systemic therapies in the context of pain relief in PNP (Haanpää et al. 2016; Vinik et al. 2016). Previous studies have shown that patients exhibiting pain catastrophizing tend to experience more side effects from systemic therapy with opioids (Jamison et al. 2013) and are more likely to discontinue therapy with gabapentinoids or antidepressants (Toth et al. 2014). Therefore, compliance with systemic therapy in patients with pain catastrophizing also appears to contribute to the poorer outcome. Since local therapy with capsaicin in this study only required one application (which did not have to be applied by the patient himself), the influence of compliance problems on the success of the therapy is very small. In summary, the lower probability of systemic side effects as well as the low influence of compliance with the single application of local capsaicin could explain the good response of patients with pain catastrophizing in our study.

Our findings are not in line with a study showing poor response of patients who exhibit high pain catastrophizing to topical amitriptyline and/or ketamine cream (Mankovsky et al. 2012), but in the study mentioned, the patients had to apply the cream themselves several times a day. Therefore, these contradictory results could possibly indicate a difference in compliance in patients with pain catastrophizing depending on the type of therapy (frequency, self‐administration). Patients might additionally benefit from the close interaction with clinic staff for capsaicin patch application. Furthermore, as in the study mentioned, topical therapies were applied by the patients themselves; the substance might not have been applied as required.

### 4.3 TRPV1 and eNOS Single Nucleotide Variants

In a fixed effects model assuming additive genetic effects, TRPV1 rs222747 presented as an interesting research target. This SNV alone explains between 13% and 26% of variation in the model.

The TRP genetic variant is localised in the ankyrin repeat domains, which is hypothesized to mediate protein–protein interactions as well as the homotetramerization of the TRPV1 channel (Xu et al. 2007).

This variant, with a minor allele frequency of 26.7%, was not only shown to cause increased expression of the channel, but also exhibited an increased response to capsaicin when compared to the wild type channel in HEK cells. In the study by Wang et al. (2016), who investigated the functional impact of TRPV1 SNVs in HEK293 cells electrophysiologically, it was shown that the SNV rs222747 causes more capsaicin‐induced desensitisation (compared to wild‐type TRPV1).

A higher response of the channel itself to capsaicin as well as higher expression levels, reflecting an increased abundance of the target structure, may lead to an increased effect of capsaicin.

Despite the sensory changes associated with it, suggesting a functional impact of the variant, rs8065080 did not appear to predict response, possibly explaining the lack of clear predictors upon quantitative sensory testing variables.

The eNOS variant investigated in this study did not show any influence on response to treatment with high‐concentration capsaicin. Due to the close interaction of TRPV1 and eNOS and the influence on endothelial health shown in animal models, this remains an interesting target for further investigation, especially considering that nerval and endothelial pathologies co‐occur in common diseases like diabetes mellitus (Castrejón‐Téllez et al. 2022; Torres‐Narváez et al. 2019).

### 4.4 Limitations

Our study does have several limitations. Firstly, this was a non‐interventional trial, recruiting patients regularly scheduled to receive high‐concentration capsaicin treatment. Therefore, patients and investigators were not blinded and the study lacked a placebo control, meaning that some of the identified predictors might in part predict the placebo effect, which we already discussed regarding pain catastrophizing. Furthermore, patients were allowed to continue concomitant pain medication according to clinical routine, which varied between patients.

Additionally, the sample size of the study was relatively small.

Therefore, the predictors might be overfitted to our specific cohort and the results might not be generalizable. The small sample size limits the diversity of our cohort. Confirmation of the results in larger cohorts or a cohort with a more diverse or different genetic background is needed.

### 4.5 Clinicial Relevance

Treatment with high‐concentration capsaicin patches is an established and effective option for patients with peripheral neuropathic pain. However, as the procedure is relatively resource‐intensive and may cause transient discomfort, careful patient selection may allow for optimising outcomes and enhance both clinical efficacy and cost‐effectiveness. While genetic testing to guide treatment decisions currently remains impractical for routine use, questionnaires can be readily integrated into everyday clinical practice. Moreover, the emergence of standardised bedside quantitative sensory testing (QST) protocols has made sensory profiling increasingly feasible, even in non‐specialised settings (Reimer et al. 2021). Together, these approaches may support a more personalised, mechanism‐based selection of patients likely to respond to capsaicin treatment.

## 5 Conclusion

Patient‐reported catastrophizing, loss of vibration and pressure pain function, as well as the single nucleotide polymorphism rs222747 in the TRPV1 gene, were associated with the prediction of the response to high‐concentration capsaicin therapy.

While some of those parameters (input of deep somatic tissues, pain catastrophizing) may be more generalizable, others (single nucleotide variant) may be a promising target for future studies.

## Author Contributions

M.S. and L.E. contributed equally and share first authorship. The study was designed by J.F. and R.B., advised by S.B., I.C. and H.B. Experiments were performed by A.D., supervised and aided by M.S., S.R. and J.G. L.E. and H.B. conducted and interpreted genetic sequencing. J.V. analysed the data. M.S., L.E. and R.B. primarily prepared the manuscript. D.K., S.B., S.R. and J.G. contributed to data analysis, interpretation and manuscript discussion. J.F., D.K., H.B. and I.C. provided substantial revisions and critical editing. All authors discussed the results, commented on the manuscript, have approved the final version of the manuscript and agree to be accountable for all aspects of the work.

## Funding

Grünenthal GmbH.

## Conflicts of Interest

M.S. has received personal fees from Sanofi, Grünenthal GmbH, Amicus Therapeutics and Akcea Therapeutics Inc. and is a consultant for Takeda Pharmaceutical. S.B. received speaker fees from Novartis, AstraZeneca, Daiichi Sankyo. J.G. reports lecturing activities for continuing education events organised by Lilly GmbH, Novartis, Abbvie, Lundbeck, Insignia, MediSage, TEVA, MedTrix GmbJ, SchwaMedico, M.D. Horizonte, CampusWebinar; travel support by Novartis, Grünenthal, TEVA, Lilly GmbH, grants from ElectroZeutica. She is chair of the ‘German Research Network on Neuropathic Pain’. J.V. has received research funding from Viatris, paid to his institution and consultancy fees from Grünenthal, AstraZeneca and Merz Pharmaceuticals, paid to him. R.B. reports the following: Grant/Research Support from EU Projects: ‘Europain’ (115007). DOLORisk (633491). IMI Paincare (777500). German Federal Ministry of Education and Research (BMBF): Verbundprojekt: Frühdetektion von Schmerzchronifizierung (NoChro) (13GW0338C). German Research Network on Neuropathic Pain (01EM0903). Pfizer Pharma GmbH, Grünenthal GmbH, Mundipharma Research GmbH und Co. KG., Alnylam Pharmaceuticals Inc., Zambon GmbH, Sanofi Aventis GmbH, Viatris. Speaker for Pfizer Pharma GmbH, Sanofi Aventis GmbH, Grünenthal GmbH, Mundipharma, Lilly GmbH, Desitin Arzneimittel GmbH, Teva GmbH, Bayer AG, MSD GmbH, Seqirus Australia Pty. Ltd., Novartis Pharma GmbH, TAD Pharma GmbH, Grünenthal SA Portugal, Grünenthal Pharma AG Schweiz, Grünenthal B.V. Niederlande, Evapharma, Takeda Pharmaceuticals International AG Schweiz, Ology Medical Education Netherlands, Ever Pharma GmbH, Amicus Therapeutics GmbH, Novo Nordisk Pharma GmbH, Chiesi GmbH, Stada Mena DWC LLC Dubai, Hexal AG, Viatris, AstraZeneca GmbH, Sandoz, Aristo, Esanum. Consultant for Pfizer Pharma GmbH, Sanofi Aventis GmbH, Grünenthal GmbH, Lilly, Novartis Pharma GmbH, Bristol‐Myers Squibb, Biogenidec, AstraZeneca GmbH, Daiichi Sankyo, Glenmark Pharmaceuticals S.A., Seqirus Australia Pty. Ltd., Teva Pharmaceuticals Europe Niederlande, Teva GmbH, Genentech, Mundipharma International Ltd. UK, Galapagos NV, Kyowa Kirin GmbH, Vertex Pharmaceuticals Inc., Biotest AG, Celgene GmbH, Desitin Arzneimittel GmbH, Regeneron Pharmaceuticals Inc. USA, Theranexus DSV CEA Frankreich, Abbott Products Operations AG Schweiz, Bayer AG, Grünenthal Pharma AG Schweiz, Akcea Therapeutics Germany GmbH, Asahi Kasei Pharma Corporation, AbbVie Deutschland GmbH & Co. KG, Air Liquide Sante International Frankreich, Alnylam Germany GmbH, Lateral Pharma Pty Ltd., Hexal AG, Angelini, Janssen, SIMR Biotech Pty Ltd. Australien, Confo Therapeutics N. V. Belgium, Merz Pharmaceuticals GmbH, Neumentum Inc., F. Hoffmann‐La Roche Ltd. Switzerland, AlgoTherapeutix SAS France, Nanobiotix SA France, AmacaThera Inc. Canada, Hoba Therapeutics, Heat2Move, Resano GmbH, Esteve Pharmaceuticals SA, Aristo, Viatris, Vertanical GmbH. L.E., A.D., I.C., S.R. and H.B. have no conflicts of interest to disclose. J.F. reports grants and research support from DFG (FO 1311/1‐1) and Sanofi and speaker fees, consultation fees, and/or travel support from Takeda, Sanofi, and Imedu Veranstaltungsmanagment outside of the submitted work.

## Supporting information


**Data S1:** ejp70200‐sup‐0001‐supinfo.docx.
