# Supplementary material for: Response to High‐Dose Topical Capsaicin in Neuropathic Pain: Absence of Deep Pain as a Predictor of Analgesic Effect
Source: Eur J Pain. 2026 Jan 9;30(1):e70200. doi: 10.1002/ejp.70200 (PMC12785507; doi:10.1002/ejp.70200)
Supplement: Supplementary file 1 — Data S1: ejp70200‐sup‐0001‐supinfo.docx. [file EJP-30-0-s001.docx]

**Supplementary Material**

# **Table S1**

| Gene | Variant | Rs Number | Primer type | Sequence (5‘-3‘) |
| --- | --- | --- | --- | --- |
| TRPV1 | c.1911A>G, I585V | rs8065080 | F  R  S | (BTN**)** GCGCTGACCAAGCTCATTACCT  TGGCCTCGGCTCTGATGA  CCGTTTCATGTTTGTCTA |
| TRPV1 | c.1103C>G, M315I | rs222747 | R  R  S | ACAAGTTTGTGACGAGCATGTA  (BTN**)** TCCCTTCTTGTTGGTGAGCT  ATGTACAATGAGATTCTGAT |

F, Forward Primer; R, Reverse Primer; S, Sequencing Primer; (BTN), biotinylated Primer

**Table S2**

| diagnosis | | n | location of VDT | location of PPT |
| --- | --- | --- | --- | --- |
| notalgia paraesthetica | Th6 | 1 | scapula | paraspinal |
| PHN | C3-Th1 | 1 | ribs | trapezoid muscle |
|  | Th3-5 | 1 |  | paraspinal |
|  | Th5 | 1 |  |  |
|  | Th7-9 | 1 |  |  |
|  | Th10 | 1 |  |  |
|  | Th9-11 | 1 |  |  |
|  | Th10-11 | 1 | anterior superior iliac spine |  |
|  | TH10-12 | 1 | posterior superior iliac spine |  |
|  | S1 | 1 | greater trochanter | biceps femoris muscle |
| PNI | upper arm, exact nerve unclear | 1 | acromion | trapezoid muscle |
|  | median nerve | 2 | radial styloid process | thenar |
|  | radial nerve | 1 |  |  |
|  | ulnar nerve | 1 | ulnar styloid process | abductor digiti minimi muscle |
|  | arm, exact nerves unclear | 2 |  | thenar |
|  | tibial nerve | 3 | medial malleolus | abductor hallucis muscle |
|  | foot, exact nerve unclear | 2 |  |  |
|  |  | 1 | metatarsophalangeal joint of the great toe |  |
|  | peroneal nerve | 1 | lateral tibeal plateau | peroneus longus muscle |
|  | lateral thigh, exact nerve unclear | 1 | greater trochanter | greatest gluteal muscle |
|  | majority of left leg, exact nerves unclear | 1 | patella | quadriceps femoris muscle |
| PNP | hands most affected | 1 | radial styloid process | thenar |
|  | feet numb, burning pain upper legs | 2 | anterior superior iliac spine | vastus medialis muscle |
|  | feet most affected | 15 | medial malleolus | abductor hallucis muscle |
|  |  | 2 | metatarsophalangeal joint of the great toe |  |

PHN, posterpetic neuralgia; PNI, peripheral nerve injury; PNP, polyneuropathy
